# Correlative analysis of immunoreactivity in confocal laser-scanning microscopy and scanning electron microscopy with focused ion beam milling

**DOI:** 10.3389/fncir.2013.00026

**Published:** 2013-02-25

**Authors:** Takahiro Sonomura, Takahiro Furuta, Ikuko Nakatani, Yo Yamamoto, Tomo Unzai, Wakoto Matsuda, Haruki Iwai, Atsushi Yamanaka, Masanori Uemura, Takeshi Kaneko

**Affiliations:** ^1^Department of Anatomy for Oral Sciences, Graduate School of Medical and Dental Sciences, Kagoshima UniversityKagoshima, Japan; ^2^Department of Morphological Brain Science, Graduate School of Medicine, Kyoto UniversityKyoto, Japan; ^3^SII NanoTechnology Inc.Chiba, Chiba, Japan; ^4^Department of Anatomy, Division of Anatomy and Cell Biology, Shiga University of Medical ScienceOtsu, Shiga, Japan

**Keywords:** focused ion beam, scanning electron microscopy, immunocytochemistry, three-dimensional reconstruction, confocal laser-scanning microscope, neural circuit, synapse, neostriatum

## Abstract

Recently, three-dimensional reconstruction of ultrastructure of the brain has been realized with minimal effort by using scanning electron microscopy (SEM) combined with focused ion beam (FIB) milling (FIB-SEM). Application of immunohistochemical staining in electron microscopy (EM) provides a great advantage in that molecules of interest are specifically localized in ultrastructures. Thus, we applied immunocytochemistry for FIB-SEM and correlated this immunoreactivity with that in confocal laser-scanning microcopy (CF-LSM). Dendrites of medium-sized spiny neurons in the rat neostriatum were visualized using a recombinant viral vector, which labeled the infected neurons with membrane-targeted GFP in a Golgi stain-like fashion. Moreover, the thalamostriatal afferent terminals were immunolabeled with Cy5 fluorescence for vesicular glutamate transporter 2 (VGluT2). After detection of the sites of terminals apposed to the dendrites by using CF-LSM, GFP and VGluT2 immunoreactivities were further developed for EM by using immunogold/silver enhancement and immunoperoxidase/diaminobenzidine (DAB) methods, respectively. In contrast-inverted FIB-SEM images, silver precipitations and DAB deposits were observed as fine dark grains and diffuse dense profiles, respectively, indicating that these immunoreactivities were as easily recognizable as those in the transmission electron microscopy (TEM) images. Furthermore, in the sites of interest, some appositions displayed synaptic specializations of an asymmetric type. Thus, the present method was useful in the three-dimensional analysis of immunocytochemically differentiated synaptic connections in the central neural circuit.

## Introduction

One of the most essential features in the functional structure of neural circuits is synaptic connection. Since synapses are small structures, they can only be analyzed using electron microscopy (EM), and thus, the three-dimensional organization of synaptic connections requires electron microscopic examination in order to reveal the design of the local neural circuit. Although transmission electron microscopy (TEM) of serial ultrathin sections has proven a useful technique for three-dimensional EM, this technique is time-consuming and requires skilled operators to cut many serial sections, capture the TEM images, align the imaging data, and reconstruct the structure (Harris et al., 2006; Hoffpauir et al., 2007). Recently, as an alternative to serial-section TEM, a novel technique has been established for the collection of serial ultrastructural images by using scanning electron microscopy (SEM). Denk and Horstmann (2004) have previously developed a serial block-face SEM (SBF-SEM) method in which plastic-embedded samples were stained with osmium tetroxide (Porter et al., 1945) and uranyl acetate (Van Harreveld et al., 1965) by using the conventional TEM method. These samples were then automatically sectioned with an ultramicrotome placed inside a scanning electron microscope column, and the block surfaces were imaged one after another by SEM to capture back-scattered electrons. The contrast-inverted images obtained by the SBF-SEM were very similar to those acquired using conventional TEM. Furthermore, serial-section SEM has been combined with the focused ion beam (FIB) milling method (Knott et al., 2008). FIB-incorporated SEM (FIB-SEM) has enabled the acquisition of three-dimensional images with a higher *z*-axis resolution compared to ultramicrotome-equipped SEM; the resolution is as thin as 4 nm using FIB milling (Knott et al., 2011) compared to 7 nm using ultramicrotome sectioning (personal communications with a reviewer). Given this high *z*-axis resolution in FIB milling, even synaptic structures parallel to the plane of the block surfaces were visualized in FIB-SEM by digitally re-slicing the stacked images through a plane that is perpendicular to the block surfaces (Merchán-Pérez et al., 2009).

Application of immunohistochemical staining for EM provides a great advantage, since functional molecules of interest are specifically localized in ultrastructures. TEM after immunostaining (immuno-TEM) for these molecules is usually applied for this purpose; however, the three-dimensional reconstruction of these images is delicate and time-consuming. Thus, it is desirable to develop a less time-consuming three-dimensional EM imaging method combined with immunostaining. Although FIB-SEM is a highly beneficial tool to acquire three dimensional data of ultrastructures, FIB-SEM has far been applied mostly to brain tissue that has been conventionally stained with osmium tetroxide and uranyl acetate. Here, we introduce the combined use of pre-embedding immunocytochemistry and FIB-SEM and demonstrate the distribution of molecules of interest in three-dimensionally reconstructed ultrastructures. Immunoreactive signals, silver grains and polymerized diaminobenzidine (DAB), were clearly visualized using FIB-SEM as applying TEM. In addition, we obtained images using confocal laser-scanning microscopy (CF-LSM; Davidovits and Egger, 1969) before the tissue was processed for EM and correlated these CF-LSM images with the FIB-SEM images. Taken together, this method may contribute to the analysis of local neural circuits at the synaptic level in the brain.

## Materials and methods

### Animals

Adult male Wistar rats (250–350 g body weight; Japan SLC, Shizuoka, Japan), were used in this study. The experiments were conducted in accordance with the guidelines of animal care by the Institute of Laboratory Animals, Faculty of Medicine, Kyoto University. All efforts were made to minimize the suffering and number of animals used in this study.

### Injection of sindbis viral vector and fixation

Six rats were anesthetized by intraperitoneal injection of chloral hydrate (35 mg/100 g body weight). To label medium-sized spiny neurons in the neostriatum, we injected 3–6 × 10^5^ infectious units (IU) of palmitoylation site-attached GFP (palGFP)-expressing Sindbis viral vectors (Furuta et al., 2001) in 1 μL of 5 mM sodium phosphate (pH 7.4)-buffered 0.9% saline (PBS) containing 0.5% bovine serum albumin (BSA) into the neostriatum (on the coronal line including bregma, 3.0 mm lateral to the midline and 4.0 mm deep from the brain surface) by applying pressure through a glass micropipette attached to Picospritzer II (General Valve Corporation, East Hanover, NJ). After 20–25 h, the rats were re-anesthetized with chloral hydrate (70 mg/100 g) and perfused transcardially with 100–300 mL PBS followed by 300 mL of 4% paraformaldehyde containing 0.05% glutaraldehyde in 0.1 M sodium phosphate buffer (PB), pH 7.4. The brains were then removed and postfixed for 4 h at 4°C in 4% paraformaldehyde in 0.1 M PB, pH 7.4. After postfixation, the brains were trimmed and cut into 50-μm-thick coronal sections by using the Microslicer (Dosaka EM, Kyoto, Japan).

### Immunofluorescence labeling and CF-LSM

The sections, including the injection site, were observed under an epifluorescent microscope (Axiophot; Zeiss, Oberkochen, Germany) to identify medium-sized spiny neurons infected with the virus. All of the following incubations were performed at 4°C followed by two 20-min washes with PBS. After incubation for 30 min with PBS containing 20% donkey serum and 0.3% Photoflo (Kodak, New York, USA), the sections with the infected neurons were incubated for 24 h with a mixture of 1 μg/mL affinity-purified guinea-pig antibody to GFP (Tamamaki et al., 2000; Nakamura et al., 2008) and 5 μg/mL affinity-purified rabbit antibody to vesicular glutamate transporter 2 (VGluT2) (Hioki et al., 2003) in PBS containing 2% donkey serum and 0.3% Photoflo (Kodak, Rochester, NY). After a rinse with PBS, the sections were incubated for 24 h with 50 μg/mL Cy5-conjugated donkey anti-rabbit IgG antibody (Merck Millipore, Billerica, Massachusetts) in PBS containing 2% donkey serum and 0.3% Photoflo. After an additional rinse with PBS, the sections were mounted onto non-coated glass slides and coverslipped with PBS.

Digital three-dimensional images were captured under a confocal laser-scanning microscope (TCS SP2; Leica Microsystems, Wetzler, Germany) using a 63 × oil-immersion objective lens (HCX PL APO CS; NA = 1.4, Leica Microsystems) with a zoom factor of 10. Native GFP fluorescence was excited using the 488-nm laser beam and observed through a prism filter set at a bandwidth of 500–550 nm; Cy5 was excited using a 633-nm beam and observed through a 640–690 nm filter. Serial TCS SP2 optical sections were obtained at an 81.4-nm step. Three-dimensional reconstruction and surface rendering of the dendrites were performed using an image processing software (Amira; Visage Imaging, Inc., San Diego, CA).

### Immunoperoxidase staining for vGluT2 and immunogold/silver enhancement for GFP

After examination using CF-LSM, the sections were further incubated with a mixture of 1:100-diluted rabbit peroxidase-anti-peroxidase (Jackson ImmunoResearch, West Grove, PA, USA), and gold particle (0.8 nm)-conjugated anti-guinea pig IgG goat antibody (Ultra Small Immuno Gold Reagents; Aurion, Wageningen, The Netherlands) in PBS containing 2% donkey serum and 0.3% Photoflo. After a rinse with PBS, the sections were washed at room temperature three times for 5 min in 0.1 M phosphate buffer (pH 7.4) (PB). The bound peroxidase developed into a brown precipitate after a 30 to 60-min reaction on ice with 0.02% DAB-4HCl and 0.0002% H_2_O_2_ in 50 mM Tris-HCl, pH 7.6. The sections were postfixed for 10 min with 1% glutaraldehyde in 0.1 M PB. After a rinse with PB, the sections were washed at room temperature two times for 5 min in distilled water, and then silver-developed in the dark with Aurion R-Gent SE-EM (Aurion). After washing, the sections were placed for 40 min in 1% OsO_4_ in 0.1 M PB, counterstained for 1 h with 1% uranyl acetate, dehydrated in an ethanol series, and flat-embedded in epoxy-resin (Luveak; nacalai tesque, Kyoto, Japan).

### FIB-SEM

**Figure 1:**
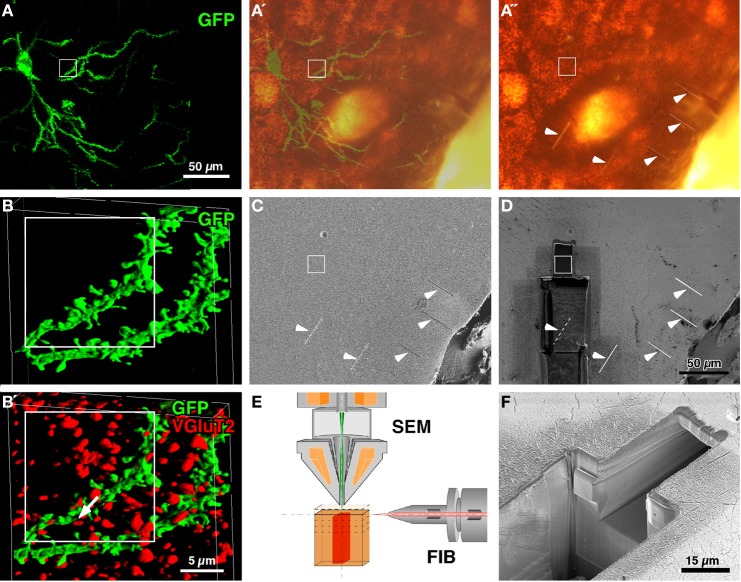
**CF-LSM images and setup for FIB-SEM.** A medium-sized spiny neuron was infected with Sindbis viral vector expressing palGFP in the rat neostriatum **(A)**. Serial digital images of a portion of the labeled dendrites were obtained, stacked (93 images at an 81.4-nm step) and reconstructed into a three-dimensional surface-rendered image **(B)**. Since the section was immunolabeled with Cy5 for VGluT2 **(B′)**, which was located at thalamostriatal terminals, many red-pseudocolored VGluT2-immunopositive terminals were in close apposition to the GFP-labeled dendrites (arrow in **B′**). After the section was developed using silver precipitation and DAB peroxidase reaction for GFP and VGluT2 immunoreactivity, respectively, the tissue was flat-embedded into an Epon block, and five short and shallow grooves were made on the block surface (arrowheads in **A″,C,D**). The silhouette of the labeled cell body and proximal dendrites in **A″** largely overlapped with the GFP fluorescence (**A′**). Using the five grooves as a landmark, the region of interest (rectangles) was approached by milling the block with FIB from the lower side **(D)**, and was examined using the FIB-SEM method (a smaller pit in **D,F**). FIB was set parallel to the block surface, and the scanning electron beam was projected perpendicular to the block surface **(E)**. **(C,D,F)**: Conventional SEM images were obtained by capturing secondary electron signals. Bar in **(D)** applies to (**A**–**A″**,**C**, and **D**); bar in **(B′)** applies to (**B** and **B′**).

After the resin was polymerized and the tissue sample was trimmed, short and shallow grooves were made on the surface of the sample with FIBs (XVision200; SII NanoTechnology, Chiba, Japan) as landmarks to link the light microscopic observation with the electron microscopic observation (Figure 1A″). To release electric charges from the sample during SEM, we coated the top surface of the sample with several 10-nm-thick layers of platinum by using a sputter coater. For three-dimensional electron microscopic analysis, the mounted sample was examined with a FIB-SEM system (SMI4000L; SII NanoTechnology). In this system, a high-resolution field-emission scanning electron microscope column was combined with a focused gallium ion beam column that was located at right angle to the scanning electron microscope column for optimized image acquisition (Figure 1E). Using an FIB of 30 kV and 700 pA, a surface layer of 20-nm thickness was conventionally milled at each sectioning. After each sectioning, the milling was paused and the freshly exposed surface was imaged in the scanning electron microscope at an acceleration voltage of 2.0 kV and beam currents of 200 pA with a dwell time of 25 μs by using the in-column energy-selective back-scattered electron detector. The retarding potential of the electron detector was set at 1.0 kV to remove secondary electrons. To increase the resolution of the *z-axis*, we further tried 5-nm-thick milling in two samples, and scanned at an acceleration voltage of 1.0 kV with a dwell time of 15 μs and at a retarding potential of 0.8 kV. The FIB gun was controlled precisely so that when an object (silicon device) with a thickness of 200 nm was cut at a step size of 5 nm, the object always disappeared between 40 and 41 steps of FIB milling. Thus, the error in FIB milling was estimated at less than 5% even with 5-nm-step milling. However, because the scanning electron beam would sometimes drift because of static electricity during SEM, it was often necessary to compensate for the drift in the position of each *x*–*y* image by using the image aligner function of the image-processing software (Amira) after image capture. TEM-like images were achieved by contrast inversion and were composed of 2000 × 2000 pixels with a resolution of 7.5 nm/pixel. The image stacks were analyzed three-dimensionally using the Amira image-processing software. For surface rendering, the contours of the immunolabeled dendrites and axon terminals were manually drawn and rendered using the Amira software.

## Results

Brain sections containing infected neurons were obtained from rats that were injected with Sindbis viral vectors expressing palGFP in the neostriatum. Several medium-sized spiny neurons were labeled with GFP fluorescence in a Golgi stain-like manner (Figure 1A) as previously reported (Furuta et al., 2001). The sections were then immunolabeled with Cy5 for VGluT2, which were reported to mostly represent thalamostriatal afferent terminals in the neostriatum (Fujiyama et al., 2006). Using CF-LSM, we acquired a three-dimensional image stack of GFP-labeled dendrites and VGluT2-immunopositive punctae (Figures 1B,B′) and determined the sites of interest, *i.e*., close appositions formed between the GFP-labeled dendrites and VGluT2-positive terminals (arrow in Figure 1B′).

To further examine the synapses using FIB-SEM, GFP, and VGluT2 immunoreactivities were developed using immunogold/silver enhancement and peroxidase/DAB methods, respectively (Figure 1A″). The neuronal silhouette of the silver precipitation was easily recognized in the flat-embedded Epon block, and the silhouette largely overlapped with the GFP-fluorescent image (Figures 1A′,A″). To localize the region for examination (rectangles in Figure 1), we made five short linear grooves on the surface of the block by using FIB (arrowheads in Figures 1A″,C,D). After making the grooves, we removed the samples from the FIB-SEM and acquired bright-field images of the samples by using a conventional light microscope to determine the positional relationship between the target regions and the grooves. Using these grooves as a landmark, we approached the site of interest by milling the block with FIB from the lower side (Figures 1D,F), and finally obtained a three-dimensional SEM image stack with a slicing thickness of 5 or 20 nm at the region indicated by the white rectangles (Figures 1C,D).

**Figure 2:**
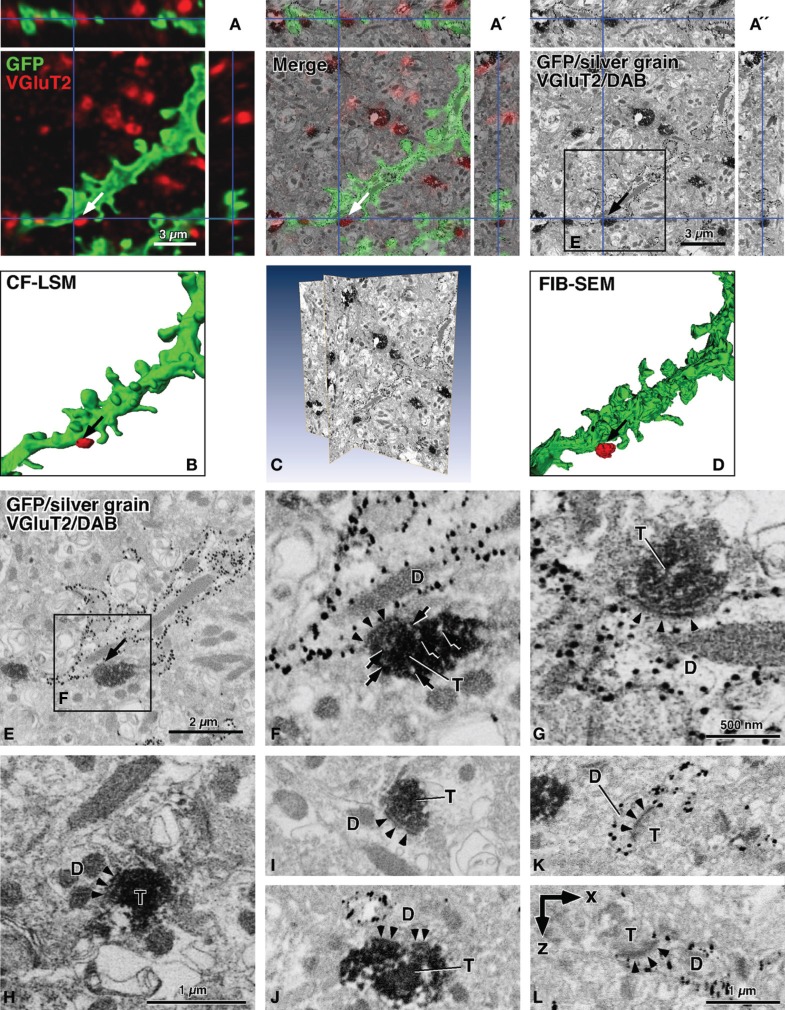
**Contrast-inverted FIB-SEM images of back-scattered electrons in reference to CF-LSM images.** In the SEM images, silver precipitations of GFP immunoreactivity were observed as fine dark grains, and DAB deposits of VGluT2 immunoreactivity were displayed as diffuse dense profiles **(A′,A″,C,E–L)**. The CF-LSM image with a resolution of 81.4 nm in the *z*-axis for GFP and VGluT2 immunoreactivities was well correlated three-dimensionally with the FIB-SEM image with a resolution of 20 nm for silver grains and DAB deposits, respectively **(A–A″)**. The surface rendering of the GFP-labeled dendrites showed that the image obtained using FIB-SEM was much finer compared to that obtained using CF-LSM **(B,D)**. The rendered image **D** was obtained after manual determination of the GFP-labeled dendritic profiles. The sites of interest (arrows in **A**–**A″,B,D,E**), where a VGluT2-positive terminal was closely apposed to a GFP-labeled dendritic shaft, showed synaptic specialization of an asymmetric type in FIB-SEM (arrowheads in **F**). Arrows in **(F)** indicate small round organelles with a pale interior, which may represent synaptic vesicles. We found no clear difference in the preservation of the ultrastructures between the long (5 min, 300 mL) **(F,G,I,K,J)** vs. short (2 min, 100 mL) **(H)** pre-rinse time with PBS before fixative perfusion. Moreover, synaptic contacts were often observed between DAB-positive terminals and unlabeled dendrites **(H,I,J)**, or between VGluT2-negative terminals and silver-labeled dendrites or dendritic spines **(K,L)**. The arrowheads in **(F–L)** indicate postsynaptic densities of asymmetric synapses, and a postsynaptic density was perforated in **(J)**. Images (**A″,C,E,F,** and **I**) were obtained using FIB-SEM at 20-nm milling steps. Images (**G,H,** and **J**–**L**) were captured using FIB-SEM at 5-nm milling steps, and image **(L)** was reconstructed in the *x*–*z* plane. By the shrinkage factor in electron-microscopic preparation, the size bar in **(A)** is slightly smaller compared to **(A″)**. D, dendritic profile; T, axon terminal. Bar in **(A)** applies to (**A** and **B**); bar in **(A″)** to (**A″** and **D**); and bar in **(H)** to (**F** and **H**); bar in **(L)** to **(I–L)**.

In Figure 2, the contrast-inverted SEM image of back-scattered electrons was three-dimensionally compared with the CF-LSM image (Figures 2A–A″), and the two images overlapped well with each other (Figure 2A′). The membrane-targeted GFP fluorescence (green in Figure 2A) corresponded with the SEM profile of the membrane structure labeled with the silver grains, which were mostly aligned on the cytoplasmic side of the membrane (Figures 2A″,E). In addition, the Cy5 immunofluorescence for VGluT2 (red in Figure 1A) correlated well with the DAB immunoreactive precipitates (Figure 1A″). Importantly, the silver grains and DAB deposits were clearly recognized in the inverted SEM images as small black grains and dense profiles, respectively (Figures 2A″,C,E–K), in the conventional immuno-TEM images. This indicated that the highly electron-dense structures obtained using the TEM technique were well-correlated with the structures that efficiently back-scattered electrons in the SEM images. Furthermore, as shown in the surface rendering of the three-dimensional reconstruction (Figures 2B,D), details in the SEM image were finer compared to those in the CF-LSM image.

Finally, using FIB-SEM, we detected synaptic specialization at the appositions that were observed using CF-LSM between the GFP-labeled dendrites and VGluT2-immunopositive terminals (arrows in Figures 1B′, 2A,A″,B,D,E). In many cases, the apposition sites formed synaptic contacts of an asymmetric type as shown in Figures 2F and G, where the arrowheads pointing to the postsynaptic density. Although synaptic vesicles in the presynaptic terminals were difficult to observe due to the intense DAB precipitates, small round organelles with a pale interior might be synaptic vesicles (arrows in Figure 2F) because the anti-VGluT2 antibody recognized the cytoplasmic portion of the synaptic vesicle membrane proteins (Fujiyama et al., 2001, 2006). In addition, silver grains representing GFP immunoreactivity were observed less frequently at the postsynaptic site compared to the extrasynaptic sites of the dendritic membrane. This might have been due to the inability of the antibody to access the antigen within the postsynaptic sites because of the steric hindrance caused by the densely and tightly packed scaffold proteins (Chatha et al., 2000). Several DAB-positive terminals in close proximity to the silver-labeled dendrites did not form synaptic contacts with the dendrites, even though there were established synaptic contacts with an adjacently unlabeled dendritic spine (not shown). In the present study, 8 (73%) of the 11 VGluT2-positive terminals that were apposed to the GFP-labeled dendrites, as seen under CF-LSM, were confirmed to make synaptic contacts with the labeled dendrites. Moreover, the three terminals that did not demonstrate a synapse formation with the GFP-labeled dendrites had established synaptic contacts with non-labeled dendrites.

Asymmetric synaptic specializations were also frequently observed between DAB-positive terminals and unlabeled dendrites (Figures 2H–J), or between DAB-negative terminals and silver-labeled dendrites (Figures 2K,L). To test how the PBS pre-rinse time before the fixative perfusion affected the preservation of ultrastructures, we compared the effect of short (2 min, 100 mL) vs. long (5 min, 300 mL) pre-rinse times. We found no obvious difference in the tissue preservation between the short (Figure 2H) and long (Figures 2F,G,I,K,J) pre-rinse times. The asymmetric synapses between the DAB-negative terminals and silver-labeled dendrites were observed mainly on the dendritic spines, most likely because the VGluT2-negative excitatory corticostriatal terminals had mainly formed synaptic contacts with the dendritic spines (for review, see Fujiyama et al., 2006). When we performed FIB milling at a *z*-axis step of 5 nm, we detected postsynaptic densities (arrowheads in Figure 2L) in the *x*–*z* (or *y*–*z*) plane, although small ultrastructures such as synaptic vesicles were not easy to observe probably because of the drift in SEM image capturing as noted in the Materials and Methods section. Taken together, we were able to examine the synapse candidates that was detected using CF-LSM at the ultrastructural level in FIB-SEM, while maintaining a good three-dimensional correlation between CF-LSM and FIB-SEM images.

## Discussion

In the present report, we showed that conventional immunocytochemical staining for TEM was applicable to FIB-SEM. Furthermore, several synaptic contacts, which were thought to exist on the basis of CF-LSM findings, were confirmed with FIB-SEM, revealing the usefulness of the combined method of CF-LSM and FIB-SEM. Although a combined method of high-resolution CF-LSM and serial-section TEM has been previously used in three-dimensional reconstruction (Dunaevsky et al., 2001), the present method enabled the reconstruction of three-dimensional images of immunopositive ultrastructures without laborious work, and the correlation of these ultrastructural images with those obtained using CF-LSM prior to EM processing.

### Immunoreactivity in FIB-SEM

The present study clearly showed that immunoreactivity with silver grains and DAB deposits appeared as dark color in contrast-inverted FIB-SEM images similar to conventional TEM images. Using FIB-SEM, the back-scattered electrons, which were produced by the projection of a tight electron beam onto the sample surface, were captured in the detector, and the image was obtained in a point-by-point manner by using the scanning electron beam. Thus, the surface point filled with the electron-scattering substance was brightly visualized. The FIB-SEM image was nearly in a reverse relationship with the TEM image since the point filled with the electron-scattering substance in the TEM sample did not transmit electrons to the detector, resulting in a dark spot in the TEM image. Metallic substances, such as silver, are well-known to scatter an electron beam, and thus, immunoreactivity with silver grains may be visualized as bright spots in the SEM image and as dark particles in the TEM and contrast-inverted SEM images.

DAB deposits do not contain any heavy metals, but osmium tetroxide is well-known to stain DAB deposits by reacting with polymerized DAB via an oxidation-reduction coupling with free amino groups in the deposits (Graham and Karnovsky, 1966). Because osmium is a highly electron-dense heavy metal, DAB deposits are electron-dense in TEM after osmium tetroxide treatment, and can be visualized as dark spots in contrast-inverted SEM images. In addition, more recent work has shown that DAB deposits are detectable in FIB-SEM (Chen et al., 2011). Thus, it is not so surprising that FIB-SEM after immunostaining (immuno-FIB-SEM) may detect immunoreactivities with silver grains and DAB deposits similar to TEM after immunostaining.

### Advantage and disadvantage of the immuno-FIB-SEM method

The proposed immuno-FIB-SEM method was useful in the three-dimensional reconstruction of immunoreactive ultrastructures, and the contrast-inverted SEM images were well-correlated with the CF-LSM images that were obtained before the sample was processed for SEM. Thus, by combining immuno-FIB-SEM with CF-LSM, the fine structures of a neural circuit in the brain, specifically synaptic connections, could be analyzed more easily compared to previous methods. However, this method exhibited the same disadvantage as the pre-embedding immuno-TEM method in which only the portion near the surface of the sections was immunostained since antibody penetration was limited in fixed tissue (Piekut and Casey, 1983). Moreover, we had to use the lowest concentration of permeabilizing reagent, such as Photoflo in this study, to increase antibody penetration. The use of detergents is known to damage ultrastructures by decreasing the contrast of membranous structures and rendering the membranes discontinuous (Wouterlood et al., 1988; Aoki et al., 1991). However, because the minimal use of a detergent enhanced antigen detectability in the conventional immuno-TEM method (Wouterlood et al., 1988; Aoki et al., 1991), many neuroscientists have applied detergent to detect and enhance the immuno-reactivity of molecules of interest (Shigemoto et al., 1997; Sugita et al., 2001; Kubota et al., 2007; Melchitzky and Lewis, 2008; Torres-Reveron et al., 2009; Chiu et al., 2010; Darcy et al., 2010; Eggan et al., 2010; Chen et al., 2012; Zikopoulos and Barbas, 2012). Aoki et al. (1991) previously reported that treatment with 0.2–0.3% Photoflo retained well-defined membranes, including synaptic organization, even though treatment with 0.06% Triton X-100 or saponin rendered most of the membrane discontinuous. Although the use of 0.3% Photoflo partially damaged membranous structures in this study, we could still detect the synaptic connections as reported in previous immuno-TEM studies. Thus, the present method was helpful in the analysis of neural circuits, which are basically formed by synaptic connections.

As a precedent case of combining CF-LSM and SEM, Micheva and Smith (2007) has described an imaging method array tomography, which has been established by applying an optical immunofluorescence technique and SEM to arrays of ultrathin, resin-embedded serial sections on glass slides. The array tomography enabled large-field volumetric imaging of immunoreactivity and ultrastructures in individual tissue specimens. Although the ultrastructures were well-preserved, and the immunofluorescence labeling was reproducible, many types of immunoreactivities might be suppressed in array tomography where the ultrathin sections were immunolabeled after osmium treatment and resin-embedding of the tissue (Van Lookeren Campagne et al., 1991). The present immuno-FIB-SEM was applicable to a large a variety of immunoreactivities as the conventional immuno-TEM. Furthermore, the *z*-axis resolution of the array tomography was limited (40–200 nm) compared to FIB-SEM (4–5 nm). Thus, despite the damage to the ultrastructures, the present method has a few advantages in addition to being less time-consuming.

Tissue information was obtained only by milling the tissue by using the FIB-SEM technique. When the region of interest was located in the center of the sample, at least one part of the sample was damaged in order to approach the target region (see Figures 1D,F). Once a hole was made using FIB, it was impossible to place a new target region in the area overlapping with the hole. Thus, when multiple regions are of interest in the sample, there is a need to design a systematic arrangement in the FIB-SEM examinations of multiple target regions. Because this arrangement could be determined in the corresponding CF-LSM images as shown in the present study, the combination of immuno-FIB-SEM with CF-LSM may be a powerful tool for the high-resolution analysis of neural circuits in the brain.

### Conflict of interest statement

The authors declare that the research was conducted in the absence of any commercial or financial relationships that could be construed as a potential conflict of interest.
